# Double QRS Transition Due to Anodal Capture During Left Bundle Branch Area Pacing: A Case Report

**DOI:** 10.3390/jcdd12080299

**Published:** 2025-08-03

**Authors:** Angelo Melpignano, Francesco Vitali, Luca Canovi, Jacopo Bonini, Ludovica Rita Vocale, Matteo Bertini

**Affiliations:** Cardiology Unit, Azienda Ospedaliero Universitaria di Ferrara, 44124 Ferrara, Italy; angelo.melpignano@unife.it (A.M.); vtlfnc@unife.it (F.V.); luca.canovi@edu.unife.it (L.C.); jacopo.bonini@edu.unife.it (J.B.); ludovicarita.vocale@edu.unife.it (L.R.V.)

**Keywords:** anodal capture, QRS transition, left bundle branch area pacing

## Abstract

Anodal capture, characterized by a different QRS morphology compared to cathodal capture, is a well-known issue in cardiac resynchronization therapy (CRT). Left bundle branch area pacing (LBBAP), a novel physiological pacing technique, is also used as a bailout strategy following failed conventional CRT implantation. In LBBAP, QRS transition, defined by a change in paced QRS morphology, serves as a key marker of successful lead placement. This case report is the first to document both high-output anodal capture and LBBAP-induced QRS transition in a single individual receiving LBBAP with an implantable cardioverter–defibrillator (ICD) as a bailout strategy for failed cardiac resynchronization therapy with defibrillator (CRT-D) implantation. Their coexistence underscores unique device optimization challenges in this emerging approach.

## 1. Introduction

Myocardial stimulation occurs due to a sudden voltage change in myocardial tissue. During bipolar pacing, this stimulation arises from the arrival of negatively charged electrons at the cathodal pole of the lead, which then return to the device via the anodal pole. The initiation of this voltage change is termed the “make” phase, and its termination the “break” phase [1].

In bipolar pacing configurations, the cathodal pole is typically programmed to the lead tip, while the anodal pole may be programmed to the lead ring, defibrillation coil (bipolar pacing), or the generator itself (unipolar pacing) [1].

Functionally, myocardial capture can occur at both poles. However, lower energy is usually required at the cathodal pole to capture tissue (except during the relative refractory period), whereas higher energy is needed at the anodal pole, particularly when the anode has a large surface area, resulting in lower charge density. Anodal stimulation often occurs during the “break” phase of the stimulus [2,3,4].

Modern devices, especially cardiac resynchronization therapy (CRT) systems, allow for multiple pacing vectors between right ventricular (RV) and left ventricular (LV) leads. For example, the cathode may be programmed to the LV lead tip and the anode to the RV lead ring to optimize thresholds [1].

When pacing the left ventricle in a bipolar configuration (LV tip [cathode] → RV ring [anode]), high pacing outputs (e.g., for patients with elevated LV thresholds) may inadvertently cause anodal capture at the RV site. This can result in RV-only pacing (due to failed LV capture), leading to loss of CRT benefits and associated clinical risks [5]. To mitigate this, reprogramming the anode to a larger surface area (e.g., defibrillation coil) reduces charge density and suppresses anodal capture [4]. For this reason, anodal capture is rarely observed with RV leads with an integrated bipolar design [6].

Left bundle branch area pacing (LBBAP) has emerged as a novel technique in conduction system pacing (CSP), addressing limitations of His Bundle Pacing (e.g., unstable electrical parameters and procedural complexity) [7,8]. It produces a physiological LV activation pattern comparable to CRT, making it a viable option as a bailout strategy during failed CRT implantations (although poorly supported by current guidelines) or for patients who do not meet CRT criteria but may benefit from physiological pacing [9].

Several electrocardiographic criteria are used to confirm successful LBBAP, as shown in Table 1 [10,11].

Among these, QRS transition (though rarely observed) has the highest specificity for confirming true left bundle branch (LBB) capture [10,11].

Transition from ns-LBBP to s-LBBP is defined by prolongation of the stimulus-to-QRS interval (measured from the pacing artifact). On the ECG, this manifests as the following:

- A rounded R’ wave in lead V1, accompanied by prolongation of the V1 R-wave peak time (V1RWPT) exceeding 10 ms.
- The development of a deeper S wave in leads V6 and I, while the V6RWPT remains unchanged.

Transition to LVSP, instead, reflects loss of LBB capture, resulting in pure LVSP. Key ECG features include the following:

- Prolongation of the V6RWPT, indicating delayed activation of the left ventricular lateral wall.
- Reduced R’ wave amplitude in V1, reflecting diminished direct LBB activation.
- Disappearance of S wave in lead I and V6, consistent with altered ventricular depolarization patterns [10].

The QRS transition during LBBAP has two key aspects impacting its reproducibility over time: (1) abrupt transition nature—in fact, the shift between ns-LBBAP to s-LBBAP morphology is intrinsically abrupt, often occurring as a single-beat transition without progressive intermediate morphologies; (2) transient capture instability—when transition occurs, it typically manifests in isolated cardiac cycles before immediate capture stabilization, making its documentation chance-dependent rather than systematic.

A novel finding is the simultaneous occurrence of anodal capture and QRS transition in a patient undergoing LBBAP with ICD backup—a bailout strategy after unsuccessful CRT-D implantation. This highlights previously underreported programming considerations in such hybrid procedures.

## 2. Case Presentation

A 75-year-old man with a history of acute myocardial infarction treated with coronary artery bypass grafting (CABG; left internal mammal artery to left anterior descending artery) and concomitant modified endo-ventricular circular plasty (Dor procedure) for apical aneurysm and atrial flutter treated with transcatheter ablation in 2022, targeting a critical isthmus at the cresta terminalis, subsequently developed sinus node dysfunction requiring permanent pacemaker implantation. Optimal medical therapy for heart failure was initiated and titrated over several months.

Transthoracic echocardiography revealed left ventricular hypertrophy, septal dyskinesia, and apical akinesia, resulting in severe left ventricular dysfunction (left ventricular ejection fraction, LVEF 25%). Given persistent LV dysfunction despite optimal medical therapy and the presence of LBBB with a QRS duration of 164 ms in a well-preserved patient with good functional status, an upgrade to CRT was indicated.

**Figure 1:**
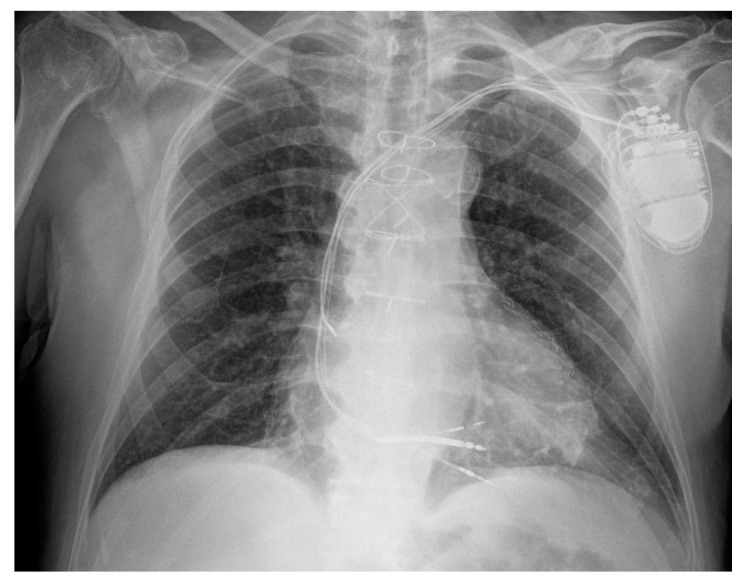
Post-procedural chest X-ray showing the implanted device, the connected leads (atrial lead, 3830 lead for LBBAP, right ventricular defibrillator lead), and the abandoned right ventricular pacing lead.

**Figure 2:**
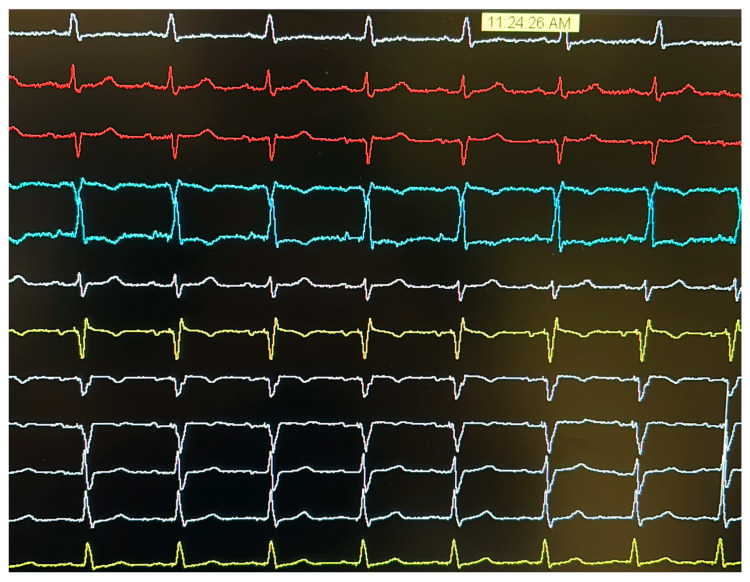
Post-procedural 12-lead ECG, showing final results at programmed output.

Pre-procedural contrast venography demonstrated occlusion of the left axillary vein; thus, the patent left subclavian vein was selected for venous access. A single-coil defibrillation lead was first positioned at the mid-apical septum of the right ventricle. Subsequent attempts to cannulate the coronary sinus for LV lead placement were unsuccessful, despite venographic guidance. As a bailout strategy, a lumenless lead (SelectSecure MRI SureScan 3830, Medtronic, Minneapolis, MN, USA) was implanted via a C315-His delivery sheath (Medtronic, Minneapolis, MN, USA) for LBBAP. The implanted device (Amplia MRI CRT-D SureScan, Medtronic, Minneapolis, MN, USA) was connected to the defibrillation lead (DF4 port) and the LBBAP lead and the pre-existing atrial lead (IS1 port). The pre-existing right ventricular lead was retained and abandoned after weighing the procedural risks associated with its removal (Figure 1). Post-procedural 12-lead ECG, showing the final result at the programmed output, recorded by the polygraph, is provided in Figure 2.

The initial LBBAP threshold was 2.0 V@0.4 ms, with QRS transition from non-selective to selective LBB capture at 2.2 V@0.4 ms. After current of injury (COI) reduction, the threshold improved to 1.0 V@0.4 ms, with QRS transition at 1.1@0.4 ms. 

The following day, threshold testing was repeated with multiple vectors to optimize pacing parameters, and the final pacing configuration entailed ventricular stimulation exclusively through the left bundle branch catheter (LV only configuration).

**Figure 3:**
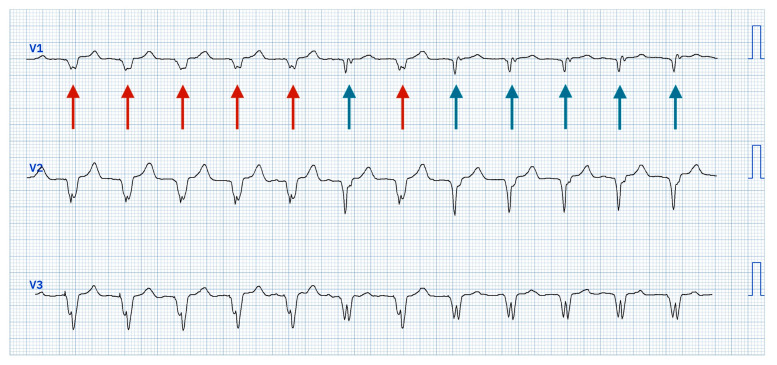
High output decremental pacing at voltage from 5 V@0.4 ms to 4.25 V@0.4 ms (auto-decrement each three beats) with initial anodal capture resulting in right ventricular apical pacing (red arrows) and then transition of QRS to non-selective left bundle branch area acing (ns-LBBAP—blue arrows).

**Figure 4:**
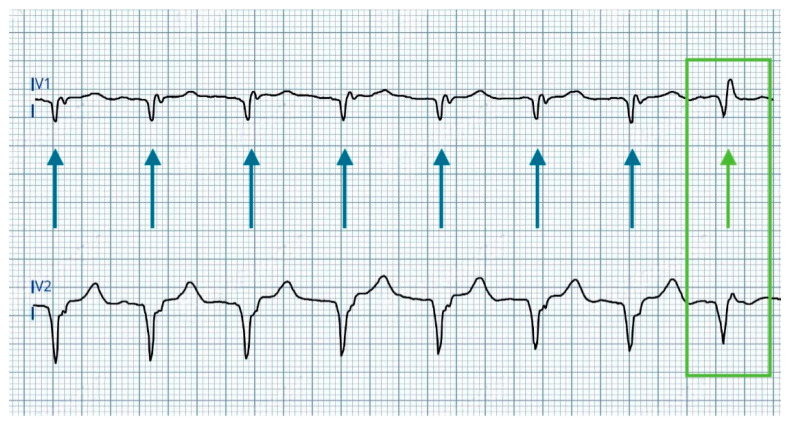
ECG during threshold tests between 1.5 V@0.4 ms and 1 V@0.4 ms showing QRS transition (last beat) from ns-LBBP (blue arrows) to selective left bundle branch pacing (s-LBBP—green arrow and box).

During bipolar pacing (LBBAP tip → RV defibrillation lead ring) starting from 5 V@0.4 ms, two distinct QRS morphology changes were observed:

1. At 4.5 V@0.4 ms: morphology consistent with right ventricular apical pacing, attributed to anodal capture at the RV defibrillation lead ring changes to a ns-LBBAP morphology (Figure 3).
2. At 1.0 V@0.4 ms: transition from ns-LBBAP to s-LBBAP, with threshold at 0.75 V@0.4 ms (Figure 4).

In summary, voltage reduction during threshold testing revealed a double QRS transition, characterized by three distinct morphologies:

- At >4.5 V@0.4 ms: dominance of anodal capture (RV apical pacing) due to high charge density at the anode.
- At 4.5 V–1.25 V@0.4 ms: ns-LBBAP due to progressive reduction in anodal interference.
- At ≤1.0 V@0.4 ms: s-LBBAP, with singular capture of the LBB.

This double transition—from anodal capture to ns-LBBAP, and ultimately to s-LBBAP—highlights a critical gradient: anodal capture dominates at high outputs, while physiological LBB capture emerges at lower voltages, underscoring the importance of optimizing stimulation parameters to achieve and maintain LBB capture for maximal clinical benefits.

Different measurements pertaining to the three distinct QRS morphologies are summarized in Table 2.

## 3. Discussion

Although the use of CRT devices has declined with the spread of LBBAP, certain challenges, well-known in CRT implantation, must also be considered for CSP.

A straightforward scenario of anodal capture at the LBBAP lead ring has recently been described. In such cases, the physiological benefits of LBBAP could be lost due to anodal capture at the lead ring; however, the hemodynamic effects remain controversial [12,13].

In the case presented here, however, the mechanism of anodal capture resembles that observed in CRT implants, where cathodal and anodal sites are located on two separate leads. This scenario must be carefully considered to prevent the loss of the benefits from both CRT and LBBAP.

The critical need to identify anodal capture in the context of LBBAP implantation is essential because, despite the significantly lower pacing thresholds compared to coronary sinus LV leads, the phenomenon described can still occur in case of automatic capture verification failure or an excessively high safety margin programmed. Therefore, awareness of this mechanism is paramount, as it subverts the goal of physiological conduction system pacing, resulting instead in unintended RV myocardial capture with consequent loss of LBBAP benefits.

To avoid anodal capture in this context, it is needed to

- Confirm that anodal capture occurs only at voltages below the programmed pacing output, ensuring an appropriate safety margin.
- Select a different anodal pole for bipolar pacing. In devices with a defibrillation lead, choosing a larger-area anode (e.g., the defibrillation coil) can reduce charge density.

## 4. Conclusions

Anodal capture, a well-known issue in CRT implants, must also be considered in the context of LBBAP, particularly in complex devices with both an RV defibrillation lead and an LBBAP lead. This is especially critical in multi-vector pacing configurations to optimize the benefits of physiological stimulation and avoid the detrimental effects of unintended RV apical pacing.

## Figures and Tables

**Table 1:** Electrocardiographic criteria used to confirm successful LBBAP implant.

Criterion	Description	Notes
QRS transition	- During threshold testing: Transition from non-selective LBBP (ns-LBBP) to selective LBBP (s-LBBP) or left ventricular septal pacing (LVSP) - During programmed stimulation: Transition to s-LBBP with pacing output adjustments	Observable with pacing output changes
V6 R-wave peak time (V6RWPT)	Time from pacing stimulus to peak of R-wave in lead V6	<75 ms if arrow native QRS or isolated RBBB <80 ms in case of LBBB, IVCD, RBBB + fascicular block, wide escape rhythm, or asystole
V6-V1 interpeak interval	Time difference between peak of R-wave in V6 and peak in V1	>44 ms
Stimulus-to-potential alignment	Concordance between: 1. Intrinsic potential-V6RWPT; 2. Stimulus-V6RWPT.	Difference ≤ ±10 ms

**Table 2:** Measurements for the three paced QRS morphologies.

	RV Anodal Capture	ns-LBBAP	s-LBBAP
Stim to peak interval (V6RWPT)	88 ms	74 ms	74 ms
V6-V1 interval	-	126 ms	142 ms
V1RWPT	-	52 ms	68 ms

## Data Availability

Data are available on request due to restrictions.

## Abbreviations

The following abbreviations are used in this manuscript:

CRT	Cardiac resynchronization therapy
LBBAP	Left bundle branch area pacing
ICD	Implantable cardioverter defibrillator
CRT-D	Cardiac resynchronization therapy with defibrillator
RV	Right ventricular
LV	Left ventricular
CSP	Conduction system pacing
ns-LBBP	Non-selective left bundle branch pacing
s-LBBP	Selective left bundle branch pacing
LVSP	Left ventricular septal pacing
V6RWPT	V6 R-wave peak time
RBBB	Right bundle branch block
LBBB	Left bundle branch block
IVCD	Interventricular conduction delay
LBB	Left bundle branch
V1RWPT	V1 R-wave peak time
CABG	Coronary artery bypass grafting
LVEF	Left ventricular ejection fraction
COI	Current of injury
